# Constructing Menopause on Sweden's Official Healthcare Platform: A Critical Discourse Analysis

**DOI:** 10.1111/scs.70194

**Published:** 2026-02-03

**Authors:** Amanda Calvin, Kerstin Erlandsson, Camilla Udo, Lise‐Lotte Franklin Larsson

**Affiliations:** ^1^ School of Health and Welfare Dalarna University Falun Sweden

**Keywords:** digital health, health equity, health literacy, menopause, qualitative research, women's health

## Abstract

**Background:**

Menopause will personally affect half the population and can cause severe symptoms and increase long‐term health risks. Despite this, gaps in menopausal knowledge and unequal access to menopausal healthcare have been identified in Sweden. Limited knowledge among women undermines informed choice and delays care‐seeking. To address these gaps, this study purports to critically examine how menopause is represented in Swedish digital health communication and to explore the implications for informed choice and equitable care. In Sweden, the Patient Act and national quality standards guarantee accessible, evidence‐based information to support autonomy and equity. It remains unclear whether these principles are met in Sweden's primary official digital healthcare platform (1177.se).

**Aim:**

To analyse how menopause is discursively constructed on Sweden's primary official digital healthcare platform (1177.se) and to examine implications for informed choice and health equity.

**Method:**

Fairclough's Critical Discourse Analysis guided interpretation of 1177.se texts on menopause across three levels: text, discursive practice and social practice. Analytical rigour was upheld through reflexive practice and theoretical triangulation, achieved via continuous dialogue among the authors.

**Results:**

Six intersecting discourses were identified: gendered minimisation, gatekeeping, risk‐framing, neoliberal individualism, heteronormativity and intersectional exclusion. These discourses oversimplify complexity, privilege selective biomedical knowledge and moralise personal responsibility, limiting autonomy and health literacy. Such patterns contradict the principles of knowledge‐based, equitable care outlined in the Swedish Patient Act and the EU Gender Equality Strategy.

**Conclusion:**

Menopause communication on 1177.se reflects systemic tensions between governance ideals and discursive realities. To fulfil policy mandates and advance equity, digital health communication must integrate precise terminology, multilingual access, balanced risk–benefit framing and inclusive perspectives.

## 1 Introduction

Globally, social inequalities in health persist despite growing commitments to universal healthcare access. These disparities are shaped by gender, education, geography and socioeconomic status [1, 2, 3, 4]. International frameworks, including the Guttmacher–Lancet Commission and the 2030 Sustainable Development Goals, explicitly call for comprehensive sexual and reproductive health and rights (SRHR) across all life stages, ensuring bodily autonomy and equitable care [3, 5]. The European Union reinforces these objectives through policies promoting gender‐sensitive healthcare and universal SRHR access [2]. To address these gaps, this study purposes to critically examine how menopause is represented in Swedish digital health communication and to explore the implications for informed choice and equitable care.

Menopause marks a significant life stage affecting over half the population and can cause symptoms that negatively impact quality of life as well as long‐term health risks such as osteoporosis, cardiovascular disease and genitourinary syndrome of menopause (GSM) [6]. Despite the aforementioned frameworks, attention to the health needs of women during menopause remains insufficient, revealing persistent gaps in inclusive health strategies on both national and international levels [7, 8]. Barber and Charles [9] identified that women's limited menopausal knowledge and fear of breast cancer were key barriers to seeking healthcare for menopausal symptoms.

In Sweden, the Patient Act guarantees patients the right to clear, evidence‐based information that enables autonomy in decision‐making [10]. This principle is reinforced by the Swedish National Board of Health and Welfare's six quality dimensions, which require care to be knowledge‐based, safe, person‐centred, effective, equitable and accessible [11]. Swedish healthcare is founded on the principle of ‘equal healthcare for all’, supported by laws and policies aimed at ensuring gender‐equal, needs‐based and universally accessible care [12]. Furthermore, the goal of universal access to SRHR is emphasised by The Public Health Agency of Sweden [13] and by national guidelines that address women's health needs throughout the life course [14]. Swedish healthcare policy increasingly emphasises ‘quality and accessible care’, shifting services from specialists to primary care providers [15]. 1177.se is Sweden's primary official digital healthcare platform. It is managed by Inera in collaboration with local regions and plays a central role in Sweden's healthcare policies. It serves as the first point of contact in primary care, supporting self‐triage where individuals choose between self‐care and healthcare services. The platform has two distinct sections: one for the public, offering health information in 21 languages, easy‐to‐read Swedish, and Swedish Sign Language, and one for healthcare professionals providing clinical guidelines and resources [16, 17]. As a trusted national source, 1177.se is a critical tool for reducing knowledge gaps and supporting informed choice.

Despite these commitments, The National Board of Health and Welfare [18] released a report identifying clear gaps in menopause‐related knowledge, signalling inadequate access to informed choice. According to their report, only 1 in 10 women in Sweden feel adequately prepared for menopause, and access to menopausal healthcare differs across sociodemographic and geographic lines. The report highlights 1177.se as a critical tool for addressing these knowledge gaps [18]. Informed choice requires access to evidence‐based knowledge, freedom from myths and misinformation, combined with easy access to care, treatment options and follow‐up. This concept originates in feminist healthcare research and is closely tied to women's right to information about complex health issues [19], in this case weighing the benefits and risks of menopausal hormone therapy (MHT) [20], making it a relevant lens for analysing health literacy in menopause care.

It has previously been described that government‐funded health communication is not neutral; language constructs health, illness and responsibility, shaping both expectations and behaviours [21]. Critical discourse analysis (CDA) provides tools to examine how power, knowledge and ideology operate through language in health information [22]. Since 1177.se is used (and trusted) by the public as well as healthcare professionals, it is important to scrutinise it. The Swedish healthcare system frequently refers the public to finding information and to follow recommendations provided on 1177.se. Given its wide reach and institutional credibility, the content holds considerable influence over public understanding of health, illness, and when to seek healthcare. By analysing how menopause is discursively represented on 1177.se, this study explores whether Sweden's commitment to equity and person‐centered care is reflected in practice. This study therefore aims to critically analyse how menopause is discursively represented on Sweden's primary official digital health platform, and to discuss the implications for informed choice and equitable care.

## 2 Method

This study employed Fairclough's three‐dimensional model of CDA, which aims to explore the relationship between language use and social practice. To achieve this, a linguistic analysis was conducted to identify discursive practices and examine their broader societal impact [22]. The model's three analytical levels are outlined in the following sections.

### 2.1 Level 1—Textual Analysis

Texts were examined for linguistic features, including interactional control, value‐laden words, frequency and modality in the construction of key concepts. The purpose was to identify linguistic strategies used to shape readers' perceptions of the intended message [22, 23].

### 2.2 Level 2—Discursive Practice

This level focused on text production and dissemination, as well as how texts were intended to be received by readers. It involved identifying power relations embedded in language and examining how texts build on, reproduce, or construct discourses [22, 23].

### 2.3 Level 3—Social Practice

Finally, texts were situated within broader socio‐cultural and institutional contexts to explore how societal power dynamics are embedded in language [22, 23].

## 3 Material

1177.se, Sweden's national digital health platform, is primarily available in Swedish though it also provides health information in Swedish Sign Language, easy‐to‐read Swedish, Arabic, English, Finnish, Jiddisch, Lule Sami, North Sami, Persian, Romani čhib arlikane, Romani chib kalderašicko, Romani chib kálo, Romani chib lovaricka, Russian, Somali, South Sami, Spanish, Tigrinya, Tornedalen Finnish and Ukrainian.

In February 2025, the first author searched the platform using the internal search function with the term *menopause*. Searches were conducted in Swedish (65 unique hits) and English (0 hits). For other languages, AI‐assisted searches were performed, yielding no additional hits. Consequently, the analysis was based exclusively on Swedish texts. All hits containing the term *menopause* were included, both those directly addressing menopause and those providing contextual information such as symptoms, treatments and associated conditions (e.g., osteoporosis, urinary problems and myomas). A second search in June 2025 confirmed that no updates had been made to the identified content.

The corpus comprised approximately 112,575 words across 65 webpages. For analysis, all paragraphs containing information and recommendations related to menopause were extracted into a structured coding document, resulting in a dataset of approximately 10,000 words.

## 4 Analytic Process

The first author conducted a close, line‐by‐line reading of the texts, focusing on vocabulary choices, modality (e.g., expressions of power) and rhetorical strategies framing menopause. Reflexive discussions and iterative analysis were conducted with the multidisciplinary research team, including a caring science perspective and a social science perspective, in line with the epistemological assumptions of CDA [22, 23].

At the level of discursive practice, attention was given to how texts were authored, edited and framed for the intended audience, identifying six dominant discourses. The social practice analysis situated these discourses within the Swedish healthcare context, characterised by universal access and value‐based care, and examined their relation to medical and political governance. This layered approach allowed the authors to move beyond descriptive content and critically analyse how medical and policy domains co‐construct language and shape public understanding of menopause [21].

For an overview of the analytical structure, see Table 1.

**TABLE 1:** Overview of analysis.

Text	Discursive practice	Social practice
Use of minimising language e.g., ‘it is common to have mild menopausal discomfort during this period’ [24], ‘symptoms usually pass’ [24]	Reinforces menopause as minor, non‐medical; simplifies diagnosis and care pathways	Reproduces gendered minimisation of female suffering; legitimises under‐treatment
Omission of statistics; selective use of scientific terms e.g., ‘It is unclear why some people have no symptoms, or why symptoms in most people decrease or go away’ [24]	Withholds context for symptoms or care; imbalanced explanation of treatment options	Maintains medical authority and control over access to care and knowledge
Emphasis on ‘risk’ without quantification; weak modality in benefit framing e.g., ‘The risk of breast cancer increases if you receive oestrogen treatment for more than 5 years’ [25], ‘MHT also reduces certain risks’ [25]	Amplifies fear over treatment (esp. MHT); discourages informed choice	Reflects medical conservatism and cultural memory of WHI controversy
Prescriptive tone e.g., ‘Physical activity is good … It reduces hot flashes, sweating and chills’ [26], General health advice as menopause care e.g., ‘'rest if you are tired’ [26]	Frames individual lifestyle as central to symptom control	Aligns with neoliberal health discourse: moralization of personal responsibility
Focus on penetrative sex, ‘help from partner’, assumed heterosexual roles e.g., ‘Some people experience decreased sexual desire during menopause. One reason may be that the vaginal mucous membranes become dry. This can make vaginal intercourse uncomfortable or painful. Fatigue and low mood can also affect sexual desire. Using a softening ointment, lubricant, or a vaginal oestrogen product may help if you are going to have vaginal intercourse’ [24]	Assumes male partners, traditional families, biological determinism in sexuality	Reinforces gender norms of females as primary caretakers and sexual limitations
Homogenous subject positioning e.g., absence of multilingual content; only Swedish available. Suggestions to ‘rest’, ‘exercise’, ‘communicate’ assumes access to time, money, an able body and a functioning support network	Frames ‘default’ menopause experience as cis, hetero, middle‐class, Swedish‐speaking, non‐disabled	Erases structural inequalities; limits inclusivity in public health information

## 5 Result

Based on Fairclough's three‐dimensional analysis, the textual, discursive and social practices will be presented and seen through the six dominant discourses identified: (1) Gendered minimisation; (2) gatekeeping; (3) risk‐framing; (4) neoliberal individualism; (5) heteronormativity and (6) intersectional exclusion.

### 5.1 Textual Analysis

The textual analysis examined the use of value‐laden language, lexical frequency and modality in the discursive construction of menopause on the 1177.se platform.

Within the healthcare professional section, five search‐hits were identified, none of which specifically addressed menopause, showing limited information to healthcare professionals. The corpus mainly consists of 60 search‐hit pages directed to the public. Across the platform's 21 language options, menopause‐related content was only available in Swedish and easy‐to‐read Swedish, highlighting a significant gap in multilingual accessibility. The most frequently occurring evaluative terms included *discomfort* (*n* = 78), *treatment* (*n* = 72) and *vagina* (*n* = 50), often appearing in clusters associated with symptoms, health concerns and hormone therapy. The term *risk* (*n* = 38) frequently co‐occurred with *treatment*, *side effects* and *breast cancer*, reflecting a predominantly medicalized framing of menopause. In contrast, terms such as *relief* (*n* = 10) and *prevention* (*n* = 3) were notably underrepresented, suggesting a limited focus on proactive or preventive health strategies. Overall, the analysis reveals consistent lexical patterns and frequency distributions that contribute to the linguistic minimisation and normalisation of menopausal experiences. This discursive framing may constrain the scope for informed choice among users engaging with the 1177.se content.

A salient feature is the dominant use of the word *discomfort* in place of the more clinically grounded term *symptoms*. Across the corpus, *discomfort* appears over three times more frequently than *symptoms*, reinforcing a tone of minimisation and positioning menopause within a framework of minor, non‐pathological inconvenience. This lexical preference downplays the potential severity of symptoms. ‘It is unclear why some experience mild discomfort, and why most symptoms pass’. ‘It is unclear why some people have no symptoms or why the symptoms for most people decrease or go away’ [24]. Furthermore, the lexical item *common* appears 40 times throughout the corpus. The frequent use of the word *common* sets normative boundaries on what is considered common, often limiting the menopausal experience to hot flashes. The texts predominantly employ vague modality, epistemic uncertainty, and reassuring tones that minimise the physical and psychological complexity of the menopausal experience.

The term *menopause* is used as a simplified umbrella term encompassing all phases of menopause, perimenopause, menopause and postmenopause. This generalisation contributes to conceptual imprecision and semantic ambiguity across the analysed texts. By collapsing the distinct phases into a single term, the text obscures important physiological and psychological differences between the phases. For example, the uterine myoma page states that myomas are more common ‘the closer you get to menopause’ [27], without clarifying that this refers to perimenopause. Later, it notes that myomas ‘usually shrink or disappear when you enter menopause’ [27], implicitly referring to postmenopause. The lack of clear distinctions between perimenopause, menopause and postmenopause risks reinforcing conceptual confusion and limiting public understanding. The failure to employ precise terminology, such as explicitly naming the perimenopausal or postmenopausal phase, reduces clinical clarity and may mislead readers about symptom progression and hormonal dynamics. Likewise, the simplified word *vagina* is used to encompass all vulva‐vaginal symptoms, thereby diminishing the complexity of genitourinary syndrome of menopause. The textual pattern across the corpus illustrates how lexical choices such as *discomfort*, the simplified language surrounding the menopausal phases, and the normalising function of the word *common* collectively reinforce a discourse that minimises menopausal complexity. Vague statements construct epistemic ambiguity through phrases such as *it is unclear* and employ softening expressions like *mild discomfort* and *will pass*, even when describing clinically significant symptoms.

### 5.2 Discursive Practice

The discursive practice focuses on the communicative event in regard to power aspects of the text such as building on, reproducing or creating discourses.

The texts on 1177.se are produced by order of the Swedish government and reviewed by medical professionals in their respective fields, which implies an authoritative relationship between the writer and the reader. The text creators construct an image of the reader by communicating according to assumptions embedded in multiple societal discourses. The text in turn then shapes the readers' understanding of menopause.

The *Gendered minimisation discourse* contributes to downplaying and normalising suffering as well as potentially diminishing the perceived legitimacy of seeking medical evaluation or support. This is reinforced by normalising statements such as ‘It is common to have mild menopausal discomfort during this period’ [24], which discursively positions menopausal symptoms as minor and expected, potentially discouraging help‐seeking. At the level of discursive practice, the language used in the 1177.se menopause‐related health texts systematically downplays the severity and complexity of menopausal experiences. This discursive minimisation reduces the perceived need for medical attention and support. The lack of specificity in defining *menopause* leads to inconsistent messaging about hormonal influences and symptom trajectories. In addition, the frequency and contextual use of the word *common* works discursively to frame a narrow range of symptoms and biological processes as predictable or routine, which may marginalise less common but clinically significant experiences. Such framing risks marginalising those whose experiences fall outside these dominant narratives, reinforcing a narrow biomedical script of menopause, and reducing their perceived need for medical intervention. Psychological symptoms are externalised or attributed to social stressors rather than hormonal changes: ‘Some may experience anxiety or depression, but this is usually due to something other than menopause’, ‘For some, menopause coincides with working and having significant family and childcare responsibilities […] This can also affect mood and sleep’ [24]. This externalisation contradicts emerging evidence linking hormonal fluctuations and decrease to psychological symptoms, suggesting that biomedical gatekeeping undermines women's biological realities by privileging sociocultural explanations.

In the Gatekeeping discourse the texts omit epidemiological data that could contextualise the prevalence and treatment needs of menopausal symptoms. Despite evidence that most women experience menopausal symptoms and that one‐third may benefit from treatment, women are not presented with information that could put their menopausal experience into context. Another aspect subjected to gatekeeping is the preventive potential of vaginal oestrogen for genitourinary syndrome of menopause as it is primarily mentioned as a remedy for immediate discomfort: ‘Over‐the‐counter estrogen medications are available at the pharmacy for genital usage. They may help if your vagina feels dry and if you experience burning or itching’ [26]. Vaginal oestrogen is further problematized in relation to the risk: ‘Because the hormones do not spread throughout the body, there is no increased risk of breast cancer, uterine cancer or blood clots’ [25]. While factually correct, this reassurance may inadvertently heighten fear in the readers of the text by reminding them of the small risks associated with systemic MHT in a generalised way.

The *Risk‐framed discourse* is communicated by highlighting potential risks, without contextualising them statistically or balancing them by presenting evidence‐based potential benefits of MHT. ‘The risk of breast cancer increases if you receive estrogen treatment for more than 5 years’ [25]. In contrast, potential benefits are noted briefly and without elaboration: ‘MHT also reduces certain risks’ [25]. This may deter women from understanding the complexity of menopausal treatment and from considering beneficial treatment while fostering the longstanding disproportionate fear.

The *Neoliberal discourse* prioritises individual responsibility through self‐care and lifestyle advice: ‘You can influence how you feel in various ways’. ‘Physical activity is good. It helps if you have difficult mood swings, feel down, or have trouble sleeping. It also reduces hot flashes, sweating and chills’ [25]. Although some lifestyle recommendations are evidence‐based, they lack necessary detail regarding frequency, duration, or feasibility. Suggestions such as ‘rest when tired’ both minimise symptom severity and question the reader's ability to understand basic self‐care strategies. The trivialization is further underscored through advice such as ‘Wear loose clothing and dress in many thin layers’ [26], which reduces a complex biopsychosocial transition to banal lifestyle adjustments, implicitly assigning responsibility to the individual rather than the healthcare system. Conflation of wellness and medical advice can be seen as both exclusionary and confusing.

The *Heteronormative discourse* presents a consistently gender normative and biologically reductive view of women. Gendered expectations are reinforced through references to caregiving implications that position women as caregivers and that domestic responsibilities are not equally shared and are instead something that their partner can help them with: ‘As a close relative, you may help in practical ways. Some examples are taking care of children or grandchildren, caring for elderly relatives, and managing the household’ [26]. Low libido is presented as a common symptom of menopause, creating an expectation of low libido and excluding diverse experiences. Communication with a partner is presented as a solution for low libido: ‘Talk to the person you have sex with about how you want it and what feels best’ [26]. In the information section aimed at family members, sexual intimacy is constructed through a heteronormative, penetration‐focused script. The partner is positioned as the sexually active subject, while the woman is framed as passive and potentially in pain, illustrated by the advice: ‘take it easy if you have sex and they experience pain’ [26]. Sexual challenges are largely constructed as problems of vaginal pain during penetrative sex, and solutions are framed accordingly. The suggestions and information primarily provide an equivalence between libido and penetrative intercourse. However, women's sexual experiences encompass far more than penetration, a perspective that is notably absent from the texts. This implicitly reinforces penetrative sex and a heterosexual relationship as the norm, excluding relational diversity.

The *Intersectional discourses* are largely absent in the corpus. Advice and information are tailored to an able, heterosexual Swedish‐speaking reader with access to relational, time and financial recourses enabling self‐care and health interventions. The corpus does not acknowledge the specific needs or experiences of neurodivergent people, or those with a chronic illness or disability, diverse sexual identities, non‐Swedish speakers, or those with financial limitations. The growing recognition of increased ADHD and autism diagnoses in midlife women is also entirely absent.

### 5.3 Social Practice

In the social practice level, the findings from the textual analysis and the discourse practice level were interpreted through the domains of the medical and political spheres in contemporary Swedish society, and how they influence access to informed choice for menopausal women in Sweden.

Sweden has made a lot of progress in equality and women's rights regarding SRHR during the last 100 years, which has created a foundation for women with access to family planning, education and financial independence throughout their lives. However, women, especially after reproductive age have been consistently sidelined. The identified discourses show a narrow construct which is an exclusionary and risk‐averse view of menopause, where Swedish women are addressed as self‐responsible, heterosexual individuals who are expected to manage complex bodily changes with minimal institutional support. This undermines the very principles of universal, equitable access to evidence‐based information and guidelines. A reorientation is needed, one that recognises menopause as a complex biopsychosocial transition, foregrounds women's diverse lived experiences, and provides women with the information and language necessary to make informed and empowered health choices. In a country where universal healthcare is a cornerstone, women deserve public health communication that does not render them invisible or privilege the already privileged.

Despite Sweden's commitment to universal healthcare access and gender equality, public health communication regarding menopause on 1177.se constructs women through a series of limiting discourses. The gendered discourse normalises suffering and downplays symptoms, reducing the perceived legitimacy of seeking support. The frequent use of the terms *common* and *discomfort* may implicitly signal that symptoms should be tolerated rather than addressed. This normalisation contributes to a culture where women may feel discouraged from voicing their concerns, leading to minimisation of their experiences. It reflects broader gendered patterns in healthcare, where women's suffering is often downplayed, reinforcing a sense of being devalued as women. When healthcare professionals encounter the same minimising language, it risks reinforcing attitudes that downplay women's symptoms, which may lead to further dismissal when individuals eventually seek help. Biomedical gatekeeping omits epidemiological data and uses imprecise terminology, obscuring distinctions between perimenopause, menopause and postmenopause. The corpus is also characterised by a selective invocation of biomedical authority. In combination with the fear‐framed risk discourse, this creates another barrier to informed choice as comprehensive understanding and treatment is discursively framed as an exception rather than a legitimate option. This contributes to conceptual confusion and reinforces narrow biomedical norms. A fear‐framed risk discourse presents treatment, especially MHT, through cautionary language, potentially deterring women from accessing evidence‐based and beneficial care. The continued fear of breast cancer stemming from the WHI study in early 2000 is still a barrier to prescriptions of MHT for both physicians and women. Although MHT carries small but statistically significant risks, the lowest dose and shortest possible treatment period is mentioned repeatedly without sufficient contextualization: ‘You can talk to your doctor to see if there is a lower dose of your medication to try’ [25]. ‘You can also reduce the dose by taking your medication at increasingly longer intervals’ [25]. These repeated recommendations reinforce a risk‐averse stance. While partly in line with national guidelines, the framing is unbalanced. The omission of positive outcomes such as improved quality of life, reduced risk of cardiovascular disease, osteoporosis, and relief from genitourinary syndrome of menopause contributes to a cautionary rhetoric that may prevent women from a comprehensive understanding of their options. Meanwhile, the neoliberal discourse shifts responsibility for menopausal wellbeing onto the individual women, framing self‐care as the primary solution. Within the Swedish sociocultural context, health communication reflects a broader discourse in which responsibility is placed on the individual rather than the healthcare provider. In contrast to systems where clinical authority guides care‐seeking, the emphasis in Sweden lies on self‐care, informed choice and person‐centred care. This shift transfers the focus of power from the professional to the individual, shaping expectations around health literacy and autonomous decision‐making. From a care‐seeking and nursing perspective, this raises important questions about equity, accessibility, and the support structures necessary to ensure that all women can meaningfully engage with their health during the menopausal transition. In this discourse, the management of menopausal symptoms becomes a matter of individual effort rather than structural support or medical treatment, reinforcing the idea that persistent symptoms result from poor lifestyle management rather than biological or structural factors. The heteronormative discourse reinforces norms of penetrative sex and asexuality in midlife, marginalising women's libido and sexual diversity. The target reader appears to be a cisgender, heterosexual individual in a communicative and supportive relationship. Hence, in terms of informed choice, large parts of the population are excluded, highlighting the need for inclusive information where gender norms are revised and female sexuality is seen through social, relational and conceptual lenses. The intersectional discourse is virtually absent, and, due to limited linguistic accessibility, only Swedish speaking women have access to the existing information. The discourse highlights how women with neuropsychiatric diagnoses, disabled individuals and non‐heteronormative identities are not addressed as readers. Socioeconomic factors are also invisible, as time and financial restrictions are not mentioned alongside self‐care recommendations. Linguistic and discursive choices contribute to a mechanism of exclusion from informed decision‐making and from the ability to participate in both recommendations and advice given. This reflects who society considers to be the ideal citizens who are given access to the information and recommendations available at 1177.se, namely Swedish speaking, middle‐class, heterosexual, cis women. But even they are prevented access to the full scope of informed choice as these discourses construct a narrow and exclusionary view of menopause. They are positioning Swedish women as individually responsible for navigating a complex transition in menopause without evidence‐based, structural, cultural, or medical support.

## 6 Discussion

This study examined how menopause is constructed discursively on Sweden's primary official healthcare platform, 1177.se. It demonstrates that discursive constructions of menopause on 1177.se have direct implications for women's health, informed choice, and equitable care. Our findings demonstrate that the understanding of menopause is influenced by discourses that restrict informed choice, despite legal and policy commitments to equity and person‐centred care that are fundamental to women's health. Our analysis identified six intersecting discourses shaping the information and guidance provided that may obstruct individuals' ability to make informed decisions about menopause. In addition, lack of information about menopause on pages intended for healthcare professionals may further undermine equitable menopausal care for women, limiting their ability to advise patients and provide evidence‐based, person‐centred care [9].

The shortcomings in menopause communication reflect broader systemic inequities in women's health, where information gaps undermine autonomy and equitable access to care. The Patient Act guarantees the right to accessible, evidence‐based information [10], while the Swedish National Board of Health and Welfare outlines six quality dimensions: care must be knowledge‐based, safe, person‐centred, effective, equitable and accessible [11]. These principles, reinforced by the European Commission's Gender Equality Strategy [2] and policy agreements between the state and the Swedish Association of Local Authorities and Regions (SKR) [14], apply across the care continuum. These requirements align with Sweden's broader democratic values of equal rights, equal care, and an inclusive society. Yet the current construction of menopause falls short, reproducing systemic inequities and contributing to women's longstanding disadvantages in post‐reproductive health. A population with low health literacy not only risks poorer health outcomes but also threatens the societal goal of broad participation [28].

### 6.1 Discursive Mechanisms and Governance

The identified intersecting discourses illustrate how language functions as a mechanism of governance in women's health, shaping what can be known, understood, and acted upon. 1177.se, Sweden's national digital health platform, is produced under political mandate and authored by medical experts, positioning it as an authoritative voice in Sweden's digital‐first healthcare strategy [29]. Language does not merely inform; it governs what can be understood and acted upon [22]. The six intersecting discourses, gendered minimisation, gatekeeping, risk‐framing, neoliberal individualism, heteronormativity, and intersectional exclusion, collectively narrow comprehensive menopausal understanding and undermine informed choice. The texts are written in a way which assumes a reader with the ability to be active, discerning, and capable of interpreting the information and its implicit assumptions. According to Michael Foucault, whose texts have been instrumental in the analysis of power dynamics, this is governmentality in practice—governing health through language that promotes self‐reliance rather than institutional accountability [30].

Imprecise and minimising language reduces women's ability to understand bodily changes and participate in decision‐making, reinforcing health‐literacy inequalities. Linguistic reduction exemplifies this. Menopause is framed as a single, homogenous event, erasing distinctions between the different phases of menopause, perimenopause practically erased despite growing evidence on its impact [31]. Words like *discomfort* replace *symptom*, while simplified anatomical language obscures conditions such as genitourinary syndrome of menopause. These choices, intended to improve readability, paradoxically undermine health literacy. Without precise terms, women lack the linguistic tools needed to understand and articulate health concerns, limiting their participation in clinical decision‐making and potentially delaying care [32].

Selective presentation of biomedical knowledge reinforces historical inequities in women's health by limiting access to evidence‐based treatments and amplifying fear‐based narratives. Gatekeeping compounds this limitation by omitting relevant data and only presenting select knowledge. MHT is framed primarily through cautionary language, while its benefits, such as improved quality of life and evidence‐based reduced risks for a number of health problems, receive minimal attention [33, 34]. Preventative benefits of vaginal oestrogen are also absent from the texts [35], an intervention that would be well suited for nursing a midwifery practice if they had access to the knowledge [36]. The risk‐framed discourse reinforces anxiety by presenting harms without contextual probabilities, contradicting national guidelines promoting individualised benefit–risk assessment [34]. These omissions reproduce patterns seen across women's health, where fear‐based risk communication has historically limited access to beneficial treatments. A recent British study identified similar challenges in menopausal healthcare, showing that the naturalisation of menopause, lack of knowledge and the fear of breast cancer were barriers to healthcare for all involved parties, women, GPs and gynaecologists [9]. This tension reflects what Foucault terms an epistemic shift: while a new evidence‐based paradigm emphasising individualised benefit–risk assessment is promoted within professional guidelines, traces of the previous episteme, shaped by the WHI controversy, remain embedded in public‐facing health information [37]. This coexistence of old and new ‘truth regimes’ illustrates how discursive change is neither linear nor uniform, producing governance contradictions in the translation of knowledge to practice [30].

By placing responsibility on individuals and excluding intersectional realities, the texts reinforce structural barriers in women's health and reproduce unequal access to care. Neoliberal individualism positions menopause as a personal management project, emphasising lifestyle advice over structural or professional support [22]. While self‐care is important, it is unclear which suggestions are evidence‐based symptom relief and which are general health recommendations. Exercise is presented with strong modality, but the actual effort needed for results is not [38], acupuncture is presented as an alternative, but the lack of evidence is not [39]. By framing menopausal wellbeing primarily as personal responsibility, the text obscures structural barriers within women's health, such as inadequate access to evidence‐based care, time constraints, and differential access to resources. The framing moralises healthy lifestyles and shifts responsibility away from healthcare systems. Intersectionality is virtually absent. Despite a multilingual mandate [16], menopause information exists only in Swedish, excluding non‐Swedish speakers and reinforcing systemic inequities. The assumptions regarding libido, penetrative sex, and gendered norms reinforce stigmas positioning the woman in an outdated heteronormative role [40, 41, 42, 43]. In combination, this constructs an ‘ideal patient’: Swedish‐speaking, cisgender, heterosexual, non‐disabled, middle‐class, with a support network. This resonates with Crenshaw's theory of intersectionality, which explains how multiple layers of marginalisation are formed when dominant narratives erase complexity [44]. These intersecting exclusions mirror broader inequities in women's health, where those with linguistic, socioeconomic, or neurodivergent challenges face disproportionate barriers to care.

### 6.2 Policy and Practice Implications

Menopause becomes a case through which broader systemic failures in women's health communication and policy implementation are made visible. The gap between governance ideals and discursive realities is clear. When communication privileges selective knowledge, simplifies complexity, and moralises responsibility, it undermines both autonomy and the policy frameworks designed to guarantee equity. To translate these findings into actionable strategies, Table 2 illustrates this governance function: discursive practices translate—or undermine—policy ideals such as those in the Patient Act and EU gender equality strategy [2, 10]. Thus, menopause becomes a case illustrating broader systemic failures in women's health communication. The gap between normative commitments and communicative reality shows that health information is a site of policy implementation, not a technical task. Discursive reforms—precise terminology, inclusive language, multilingual access, and balanced risk framing—are governance imperatives for equity.

**TABLE 2:** Discursive mechanisms, policy gaps and practical implications.

Discursive mechanism	Policy gap	Practical implication
Gendered minimisation	Breaches *Patient Act* and principles of knowledge‐based care	Introduce precise terms (e.g., perimenopause) with clear explanations
Gatekeeping	Misalignment with evidence‐based care	Include relevant data and medical information
Risk framing	Contradicts guidelines on individualised assessment	Balanced risk–benefit framing for MHT and alternatives
Neoliberal individualism	Conflicts with person‐centred care; risks moralising health	Present lifestyle advice as supportive, not primary; ensure structural pathways
Heteronormativity	Breaches equity and inclusivity principles in Swedish/EU policy	Use inclusive language reflecting gender and sexual diversity
Intersectional exclusion	Violates universal access mandates	Provide multilingual content and address disability and socioeconomic barriers

*Note:* The table summarises discursive mechanisms identified in the analysis and links them to relevant policy frameworks [10, 11, 12, 14].

Improving menopause communication is essential to strengthening women's health throughout the course of their lives and reducing inequities in access to evidence‐based care. Aligning health communication with policy commitments requires moving from discursive limitations toward inclusion. These suggested reforms are not merely linguistic; they are governance imperatives. Implementing them would operationalise the Patient Act, strengthen health literacy, and promote equity across Sweden's universal healthcare system [10, 12]. Language, when used inclusively and precisely, becomes a tool of empowerment, enabling autonomy and access to informed choice regarding menopausal health. On 1177.se, a narrow range of symptoms are acknowledged, just enough to demand individual action, but rarely enough to justify collective or systemic support. This exclusion is not incidental; it is structural and sustained through what Fairclough calls *discursive silence*: the omission of perspectives is itself a powerful act [22]. Such structural silence mirrors broader neglect of women's health beyond reproductive years, contributing to delayed diagnosis, fragmented care pathways, and inequitable outcomes. These challenges apply broadly but weigh heaviest on individuals outside the majority norm such as those with lower socioeconomic status and limited Swedish language skills [32]. Improving menopause communication on 1177.se therefore has direct implications for strengthening women's health throughout the course of their lives. Achieving equity in the information provided on 1177.se could be a critical first step toward closing the menopausal knowledge gap identified in the report from The National Board of Health and Welfare [18] and improving women's health beyond reproductive years.

## 7 Strengths and Limitations

This study was grounded in principles of gender equity, informed choice, and intersectional, woman‐centred care. The first author's professional background in nursing and sexual and reproductive health, along with the interdisciplinary expertise of the research team with caring perspective and social perspective, shaped the interpretative process.

Following the epistemological foundations of Critical Discourse Analysis, researchers acknowledged subject positions as analytical resources rather than biases [21, 22]. Recognising language as inherently non‐neutral, the analysis addressed how silences, normalisations, and omissions shape access to care. Reflexive dialogue ensured transparency and minimised over‐interpretation [21]. Credibility was further strengthened through analyst triangulation, drawing on interdisciplinary perspectives from caring and social sciences [45]. Interpretations remain contingent on readers' preconceptions; thus, this is one of many possible readings.

## 8 Conclusions

These findings have important implications for clinical nursing practice. Nurses often serve as the first point of contact in primary care and play a key role in translating health information into individualised care plans. This study demonstrates that menopause information on 1177.se employs discourses that constrain informed choice, undermining legal and policy commitments to equity and person‐centred care. Linguistic minimisation, gatekeeping, and risk‐oriented narratives intersect with heteronormativity and intersectional exclusions, limiting menopausal health literacy and autonomy. These patterns conflict with obligations under the Patient Act and European Commission's Gender Equality Strategies [2, 10]. Addressing these gaps requires structural reform, as outlined in Table 2, introducing precise terminology, ensuring multilingual access, and embedding intersectional perspectives. Language is not neutral—it governs care access—and must be transformed into an instrument of empowerment in universal healthcare.

## 9 Implications

The discursive patterns identified in this study have direct implications for clinical practice and the design of digital health information. Future research should evaluate whether interventions that introduce precise terminology, balanced risk framing, and inclusive language on platforms like 1177.se improve women's comprehension, engagement, and confidence in managing menopausal symptoms. Studies are particularly needed to explore how exposure to selective, minimised, or heteronormative information shapes patient guidance and constrains informed choice. Intersectional perspectives must be prioritised to address barriers faced by non‐Swedish speakers, neurodivergent individuals, those with diverse sexual identities, and people with limited socioeconomic resources, reflecting the structural exclusions highlighted in the analysis. Mixed‐methods research linking discourse analysis with user experience and outcome measures can provide concrete evidence of how discursive reforms affect autonomy, health literacy, and equitable care, and inform inclusive digital health strategies aligned with the Patient Act and EU Gendered equality strategies.

## Author Contributions

A.C. was responsible for conceptualization, data collection, primary analysis, and drafting the original manuscript. K.E. and C.U. contributed to the conceptualization and design of the study. L.‐L.F.L. was invited to the project due to her expertise in critical discourse analysis and contributed methodological guidance to the analytic process. After the initial analytic draft, A.C. worked closely with L.‐L.F.L. and K.E. to deepen the analysis and substantially revise and improve the manuscript. C.U. provided critical feedback on the final manuscript. All authors approved the final version of the manuscript.

## Funding

The authors have nothing to report.

## Ethics Statement

The authors have nothing to report.

## Conflicts of Interest

The authors declare no conflicts of interest.

## Data Availability

The authors have nothing to report.
